# Cases

**Published:** 1867-12

**Authors:** J. W. Thompson

**Affiliations:** Paducah, Ky.


### SELECTIONS.

---

*Cases by* J. W. Thompson, M. D., of Paducah, Ky.

In the August number of the *Southern Journal of Medical Sciences*, is reported a rare disease of bone, by a resident student of the Charity Hospital, New Orleans, which was treated by the visiting surgeon.

I regard the following case as the same type of disease, and like the one just referred to, as characterized by some points of pathological interest.

Miss Sallie J——, aged thirty-nine years, enjoyed usual good health to puberty. From that time, her health became impaired. She suffered with derangement of the menses, which was followed by obstinate hysteric.

From puberty until she was thirty-one years old, at which time she became my patient, she had been treated by a number of medical gentlemen, but the catamenial difficulty and hysteria continued unabated. In 1858, I first saw her, in connection with my preceptors, Drs. George Stovall and E. W. Woodson; we treated the case together, but only succeeded in giving her temporary relief.

The following year, 1859, an excrescence appeared on the middle finger of the right hand, near the distal extremity, which rather rapidly became a fungous growth, extending up the finger, despite constant local and general treatment. When it reached near the second joint, I amputated the finger. Soon after the stump became involved, and when the disease had very nearly extended to the metecarpo-phalangeal articulation, I amputated at that joint, assisted in those operations by Dr. Stovall.

Soon the index finger of the other hand—the left—took disease in the same way, a bleb appearing near the edge of the nail. In a few days, the second, or middle finger, was attacked in the same manner, the disease pursuing a similar course. When it had very nearly extended to the metecarpo-phalangeal articulations, Dr. S. S. Payne, of this county, amputated both fingers at that joint. I assisted him.

In about two and a half months from the time that operation was performed, the little finger of the same hand was affected in precisely a similar manner to those which had been amputated. She then consulted Dr. D. D. Thompson, of this city, and became his patient. Dr. Thompson informs me that, after using various local and general remedies, mentioning, especially, acid nitric and lunar caustic, the disease continued extending until it reached near the articulation of the finger with the hand, when he amputated it. Before removing that finger, Dr. T. made an incision in it and the ring finger, about an inch and a half long, extending down to the bone. After this course was pursued by Dr. Thompson, she returned to her home in Ballard county, and in about three months, the ring finger, which was the only remaining one on that hand, was affected. When it had involved almost the entire finger, I removed it at the same joint that I had done the others on that hand. In a short time after the last finger was amputated, the stump and hand became involved. Also, the left leg over the region of tibia. The leg sloughed considerably. When in this condition, setons were freely inserted in left arm and leg. Issues were kept up for two years, in which time the disease was held in check. At times, during that period, the setons were discontinued, and when that was done, the disease would return. When the issues would be again established, it would be arrested.

In this condition, she applied to a "Faith Doctress." Soon after that time, she began to improve. The knife and issues had succeeded in arresting the disease, and when the favorable change was taking place, she unfortunately, for humanity and the profession, passed into the hands of the old woman. Patients suffering with like diseases have pursued a similar course, much to the regret of the profession, because many a good person has been sacrificed by the willful imposter, through the influence of their examples.

I bring this case before the profession for the reason that I consider it exceedingly rare and interesting—interesting in both a pathological and surgical point of view.

The history of the family is not such that we could pronounce it scrofulous necrosis of the bones, and if the necrosis is of a syphilitic character, it was inherited. We admit that our pathological knowledge of that type of disease did not enable us to distinguish it.

The knife did much good in checking the disease for a time, but the setons seemed to have been more efficient than the knife. I have before stated that she enjoyed good health to puberty, but failed to make a healthy transformation from girlhood to womanhood. From puberty, the catamenia has been very irregular. For the past few years, it has occurred about semi-annually. She is a confirmed hysterical subject. The disease, on all of its fingers, first made its appearance near the distal extremity of the finger. Dr. D. D. Thompson removed the nail of one finger, but it had no influence on the progress of the disease. After removing five fingers, the disease attacked the stump of the left hand and left leg.

Issues were kept up by setons for two years, after which time the morbid condition gradually yielded. I am satisfied that the persistent use of the setons relieved the patient. It is now two years and a half since the disease disappeared, with no inclinations of its returning. I think it *possible* that had the setons been timely used the fingers might have been saved, though the medical men who were associated with me in the case, agreed with me that it was best to amputate them. In a similar case I would first thoroughly try the setons. Dr. S. S. Payne advised to establish the issues. The result of the case proved his suggestion to have been a wise one. During the time each finger was diseased, her jaws became locked and remained so for several days—at one period for eighteen days, during which time she took no food or water—bowels did not move or urine voided, except by the aid of the catheter. Did not perceptibly lose any flesh. During each spell of lock-jaws, there was paralysis of the left side of the body.

In the case reported in the Southern Journal of Medical Sciences, the disease pursued almost exactly the same course that it did in the one I have just detailed. In that case the toes were first involved by the appearance of a small bleb or blister; afterwards the phalanges became necrossed, the disease resisting all treatment, and was only temporarily checked by amputating a toe, and so on until all the toes were removed. After the disease had pursued that course, it attacked the fingers, first on the right hand, by the appearance of a bleb on the little finger, then on the left hand. Several of the fingers were amputated and diseased bones removed. When the stump of the left hand healed, the disease attacked the metatarsal bones of the left foot. The writer states that it still persisted at the time he made the report. In that case after the wound from the amputation healed, the disease remained dormant from six weeks to two months; it was the same with my patient. For more than two years there has been no recurrence of the disease. That is due, I am satisfied, to the use of the setons, for it never was dormant much longer than two months before the setons were inserted.

Some of the general symptoms that characterize my case were not present in the one referred to, but the pathological condition, I am satisfied, is the same.

Case II. Miss Susan Godsey, known as the sleeping woman. Her home is in Obion county, Tennessee.

A few days ago, I visited her and made notes of her case from observation, and the history, as given me by her mother and brother-in-law, who witnessed it from the beginning of this peculiar condition. Age twenty-seven years; has been asleep between eighteen and nineteen years; awakes almost at the *same time at certain hours each day; remains awake only from five to ten minutes;* awakes at three o'clock, A. M., then at six, and every successive hour until twelve; again awakes at three, six, seven, nine and eleven, P. M.

Pulse obscured by constant tremor of muscles; bowels generally operate once a day; menstrual period very irregular, frequently fails to appear, and when it does is generally very scanty. When awake, complains of pain in the head and down the spinal cord; the first six years, awoke only twice a day; since that time, she awakes more frequently, but for the last five years, the frequency in waking has not increased; has what I term a general spasm about every ten minutes; had two during the first twenty minutes after I saw her. Almost immediately following the spasms, she has a spell of hiccoughs, hiccoughing generally ten times each spell, at which times the head and neck are raised, and the head falls forward; awoke one time during the hour I was with her, and remained awake seven minutes; talked rationally in answer to my questions. In an instant, I discovered she had fallen asleep. At the same time, I endeavored to arouse her, but could make no impression upon her; she slept on.

It is impossible to arouse her until her usual time for waking; complains severely of cramping in the stomach and pain in left side, color is rather healthy; expression of eye good. When awake she is always gloomy; *never calls for food* but generally relishes it when given; occasionally calls for water. The mother and brother-in-law state that just before she passed into this condition, she was sick with Intermittent Fever, and that her Doctor gave her large doses of morphine, laudanum and ether, that the Doctor (?) remarked to them (the father, mother and brother-in-law,) "I have tried to cure her and failed, and then tried to kill her and failed."

The spine has been blistered and the galvanic battery used. She is medium size, moderately well developed, except the mammæ which can scarcely be said to be developed at all, and the finger and toe nails have not grown any since she passed into this condition.

This patient has actually been in a deep sleep between eighteen and nineteen years, with the exception of the few minutes each day at the stated time.

I am satisfied that this is a very remarkable case, and I have reported it for the reason that I believed the profession would be interested in the history of such an anomalous one.

I will not attempt to account for her sleepy condition. I do not feel competent to advance an opinion about a cause that is involved in such profound mystery. The sensible answers to questions and conversation while awake, make it evident that the cerebrum is not organically diseased. The difficulty may be at the base of the brain. The characteristic pain in the left side, and cramping in the stomach, attended with a rational condition of the mind, reminded me that it might be an aggravated form of the protean malady, hysterics.

I have given the mother and brother-in-law's statement of the treatment that was used immediately before she passed into this condition, for what it is worth.

What I have said about the probable cause of her condition is mere conjecture. Last spring, my friend, Dr. B. Marshall, of Woodville, furnished me the notes of her case from observing it one day; also our highly respected and intelligent Presiding Elder, Rev. Mr. Binum. These notes correspond with those made by myself. Dr. Wilson, of Union City, and Dr. Wright, of Kingston, Tennessee, were with me when I made the notes I have given in this report.

If this case has any parallel in the recorded history of medicine, I am unaware of it.

Should any one doubt the authority of this report, it can be established by numbers of the most prominent men in Western Tennessee and Kentucky.—*Nashville Journal of Medicine and Surgery.*

